# The Association of Acetabulum Fracture and Mechanism of Injury with BMI, Days Spent in Hospital, Blood Loss, and Surgery Time: A Retrospective Analysis of 67 Patients

**DOI:** 10.3390/medicina60030455

**Published:** 2024-03-09

**Authors:** Rafał Wójcicki, Tomasz Pielak, Piotr Marcin Walus, Łukasz Jaworski, Bartłomiej Małkowski, Przemysław Jasiewicz, Maciej Gagat, Łukasz Łapaj, Jan Zabrzyński

**Affiliations:** 1 Department of Orthopaedics and Traumatology, Faculty of Medicine, J. Kochanowski University in Kielce, 25-001 Kielce, Poland; 2 Department of Orthopaedics and Traumatology, Faculty of Medicine, Collegium Medicum in Bydgoszcz, Nicolaus Copernicus University in Toruń, 85-092 Bydgoszcz, Poland; 3 Department of Urology, Oncology Centre Prof. Franciszek Łukaszczyk Memorial Hospital, Bydgoszcz, 2 dr I. Romanowskiej St., 85-796 Bydgoszcz, Poland; 4 Department of Anesthesiology, Faculty of Medicine, Collegium Medicum in Bydgoszcz, Nicolaus Copernicus University in Toruń, 85-092 Bydgoszcz, Poland; 5 Department of Histology and Embryology, Faculty of Medicine, Nicolaus Copernicus University in Torun, Collegium Medicum in Bydgoszcz, 85-067 Bydgoszcz, Poland; 6 Faculty of Medicine, Collegium Medicum, Mazovian Academy in Płock, 09-402 Płock, Poland; 7 Department of General Orthopaedics, Musculoskeletal Oncology and Trauma Surgery, University of Medical Sciences, 61-701 Poznan, Poland

**Keywords:** acetabulum, fracture, pelvis, BMI, transfusion

## Abstract

*Background and Objectives*: The objective of this retrospective study was to investigate the association between acetabulum fractures; the mechanism of injury; and variables such as BMI, duration of hospital stay, blood loss, and surgery time. By exploring these factors, we aim to enhance our understanding of them and their impact on the healing process and the subsequent management of pelvic fractures. *Materials and Methods*: This study included 67 of 136 consecutive patients who were admitted for pelvic ring fracture surgery between 2017 and 2022. The data were collected prospectively at a single trauma center. The inclusion criteria were acetabulum fractures and indications for operative treatment. The exclusion criteria were non-operative treatment for acetabular and pelvic ring fractures, fractures requiring primary total hip arthroplasty (THA), and periprosthetic acetabular fractures. Upon admission, all patients underwent evaluation using X-ray and computed tomography (CT) scans of the pelvis. *Results*: The present study found no statistically significant differences between the examined groups of patients with pelvic fractures in terms of BMI, surgery duration, length of hospital stay, and blood transfusion. However, two notable findings approached statistical significance. Firstly, patients who experienced a fall from height while sustaining a pelvic fracture required a higher number of blood transfusions (2.3 units) than those with other mechanisms of injury which was close to achieving statistical significance (*p* = 0.07). Secondly, patients undergoing posterior wall stabilization required a significantly lower number of blood transfusions than those with other specific pelvic injuries (0.33 units per patient), approaching statistical significance (*p* = 0.056). *Conclusions*: The findings indicated that factors such as BMI, time of surgery, blood loss, and the duration of hospital stay were not directly correlated with the morphology of acetabular fractures, the presence of additional trauma, or the mechanism of injury. However, in the studied group, the patients whose mechanism of trauma involved falling from height had an increased number of blood transfusions compared to other groups. Moreover, the patients who had surgery due to posterior wall acetabulum fracture had decreased blood transfusions compared to those with other Judet and Letournel types of fractures. Additionally, they had the shortest duration of surgery.

## 1. Introduction

Pelvic fractures pose significant challenges for orthopedic surgeons, requiring extensive knowledge and experience to navigate safely among the organs, vital vessels, and nerves located in the pelvic bone area [1,2,3,4]. The surgical treatment of these fractures is complicated by the frequent occurrence of additional injuries, many of which can be fatal [5,6,7,8,9]. This is primarily due to the high-energy nature of these traumas [10,11,12,13,14]. Given these challenges, it is crucial to identify and manage concomitant injuries, consider the mechanism of trauma, and take into account personal details such as body mass index (BMI) to ensure appropriate treatment.

Previous studies have demonstrated that obese patients have higher rates of complications and longer hospital stays following pelvic fractures [15]. However, conflicting results exist regarding the association between decreased BMI and increased failure of internal fixation. Additionally, the duration of hospitalization after sustaining a pelvic fracture can vary significantly, with a time period of a few days to several weeks [16,17,18].

The aim of this retrospective study was to investigate the association between acetabulum fractures; the mechanism of injury; and variables such as BMI, duration of hospital stay, blood loss, and surgery time. By exploring these factors, we aim to enhance our understanding of these accompanying factors of pelvic fractures and their impact on the healing process and the subsequent management. 

## 2. Materials and Methods

### 2.1. General Characteristics

This study included 67 of 136 consecutive patients who were admitted for pelvic ring fracture surgery between 2017 and 2022. Patients included in the study who suffered from acetabular fractures qualified for operative treatment. The data were collected prospectively at a single trauma center. All patients underwent operative treatment using De Puy Synthes implants for pelvic fixation. Upon admission, all patients underwent evaluation using X-ray and computed tomography (CT) scans of the pelvis.

Demographic data were collected, including sex, age (in years), body mass index (BMI), date of injury, type of fracture, mechanism of trauma, concomitant trauma in other regions and the pelvic ring, date of surgery, surgical approach with stabilization methods, surgery duration, blood transfusions, and number of days spent in the hospital. Acetabulum fractures were classified according to the Letournel and Judet system (A+T—anterior column with posterior hemi-transverse fracture, AC—anterior column, BC—both column, PC—posterior column, PC+W—posterior column+posterior wall, PW—posterior wall, T—transverse, and T+P—transverse with posterior wall fracture), while pelvic ring fractures were classified according to the Young and Burgess system (LC—lateral compression, APC—anterior–posterior compression, and VS—vertical shear). The surgical approaches used for acetabulum fractures were the ilio-inguinal and Kocher–Langenback approaches. The ilio-inguinal approach was mainly used in the anterior wall and anterior column fixation. In the posterior wall and posterior column fixation, the Kocher–Langenback approach was mainly used. In the case of one patient with anterior and posterior wall/column fractures, both approaches were used during a single operation. 

### 2.2. Inclusion Criteria

The inclusion criteria were acetabulum fractures and indications for operative treatment, namely instability (hip dislocation associated with posterior wall or column displacement and anterior wall or column displacement) and incongruity (fractures through the roof or dome; displaced dome fragment; transverse or T-type fractures; both column types with incongruity; retained bone fragments; soft-tissue interposition). Patients who suffered from acetabular fractures with intra-articular displacement <3 mm were initially qualified for conservative treatment. The exclusion criteria were non-operative treatment for acetabular and pelvic ring fractures, fractures requiring primary total hip arthroplasty (THA), and periprosthetic acetabular fractures. 

### 2.3. Ethics

The study was conducted in accordance with the Declaration of Helsinki guidelines for human experiments. Prior to the study, permission was obtained from the local Bioethics Committee (approval number KB 645/2022). Written informed consent was obtained from all patients or their relatives upon admission to the hospital to include them in scientific studies.

### 2.4. Statistical Analysis

All group comparisons and statistical analyses were conducted by two independent investigators using Prism 9 software (GraphPad). A *p*-value of less than 0.05 was considered statistically significant. Nominal variables were described by the number of observations and their distribution. The normality of variables was assessed using the Shapiro–Wilk test. Relationships between the studied parameters were evaluated using Spearman’s rank correlation coefficient. Non-parametric tests, such as the Mann–Whitney U test and analysis of variance, were used to compare the data.

## 3. Results

Out of the initial 136 patients who underwent operative treatment for pelvic ring fractures between 2017 and 2022, a total of 67 patients fulfilled the inclusion criteria and were included in this study. The inclusion criteria required patients to have an acetabulum fracture, either with or without concomitant pelvic ring injury.

The studied cohort had a mean body mass index (BMI) of 26.25 (ranging from 18 to 39). The mean duration of surgery was 153 min, ranging from 60 to 270 min. The average number of blood transfusions received was 1.58 units, with a range of 0 to 5 units. The mean length of hospital stay was 5.25 days, ranging from 2 to 34 days.

**Figure 1:**
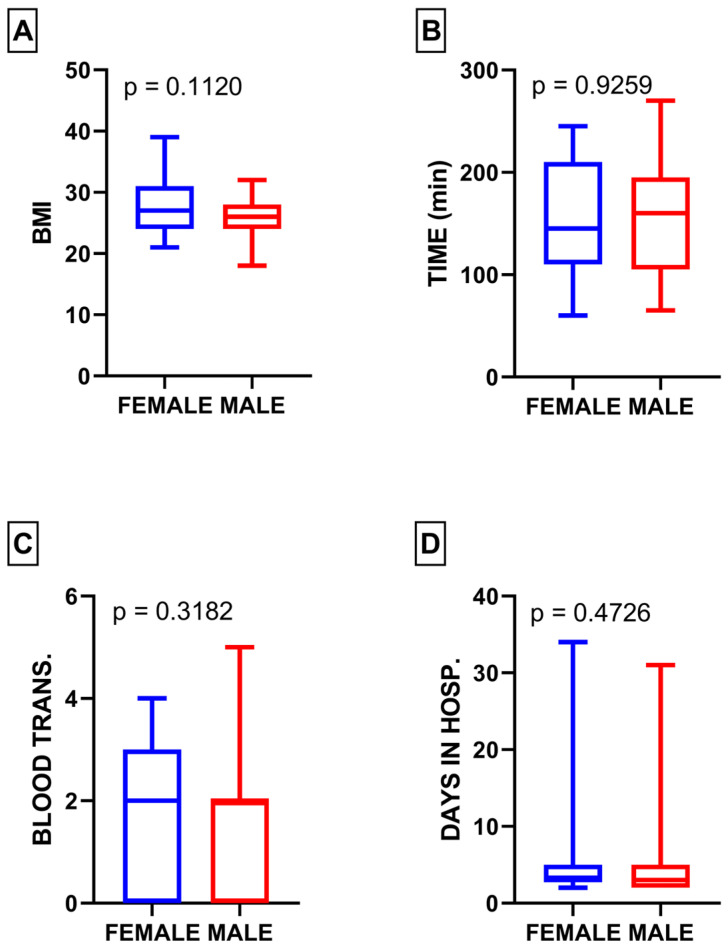
(**A**) Comparison of BMI between male and female subgroups; (**B**) comparison of surgery duration between male and female subgroups; (**C**) comparison of blood transfusion between male and female subgroups; (**D**) comparison of length of hospital stay between male and female subgroups.

In the study cohort, 15 patients (22.3%) were female, and 52 patients (77.6%) were male. Statistical analysis showed no significant differences between male and female populations in terms of BMI (*p* = 0.11), duration of surgery (*p* = 0.92), blood transfusion (*p* = 0.31), and length of hospital stay (*p* = 0.47) (Figure 1A–D).

In the female subgroup, the mean BMI was 27.87 (ranging from 21 to 39), while in the male subgroup, it was 25.79 (ranging from 18 to 32). The average surgery duration for females was 152 min (varying between 60 and 245 min), and for males, it was 153.5 min (range of 65 to 270 min). The mean length of hospital stay for females was 7.26 days (ranging from 2 to 34 days), whereas for males, it was 4.67 days (ranging from 2 to 31 days). Additionally, the mean amount of blood transfused for females was 1.86 units (ranging from 0 to 4 units), while for males, it was 1.5 units (ranging from 0 to 5 units).

The participants were categorized into various groups based on the mechanism of injury and the type of fracture according to the Judet and Letournel classification (Table 1 and Table 2). Within the studied population, there were 3 patients with anterior column with posterior hemi-transverse fractures, 14 patients with anterior column fractures, 11 patients with both column fractures, 10 patients with posterior column fractures, 4 patients with posterior column and posterior wall fractures, 6 patients with posterior wall fractures, 10 patients with transverse acetabulum fractures, and 9 patients with transverse fractures with posterior wall involvement.

The mechanism of injury presented a diverse range, with 16 patients experiencing falls from height, 3 patients involved in industrial accidents, 10 patients experiencing falls from standing height, 2 patients with pedestrian injuries, 27 patients involved in traffic accidents, and 9 patients with an unknown mechanism of injury.

**Figure 2:**
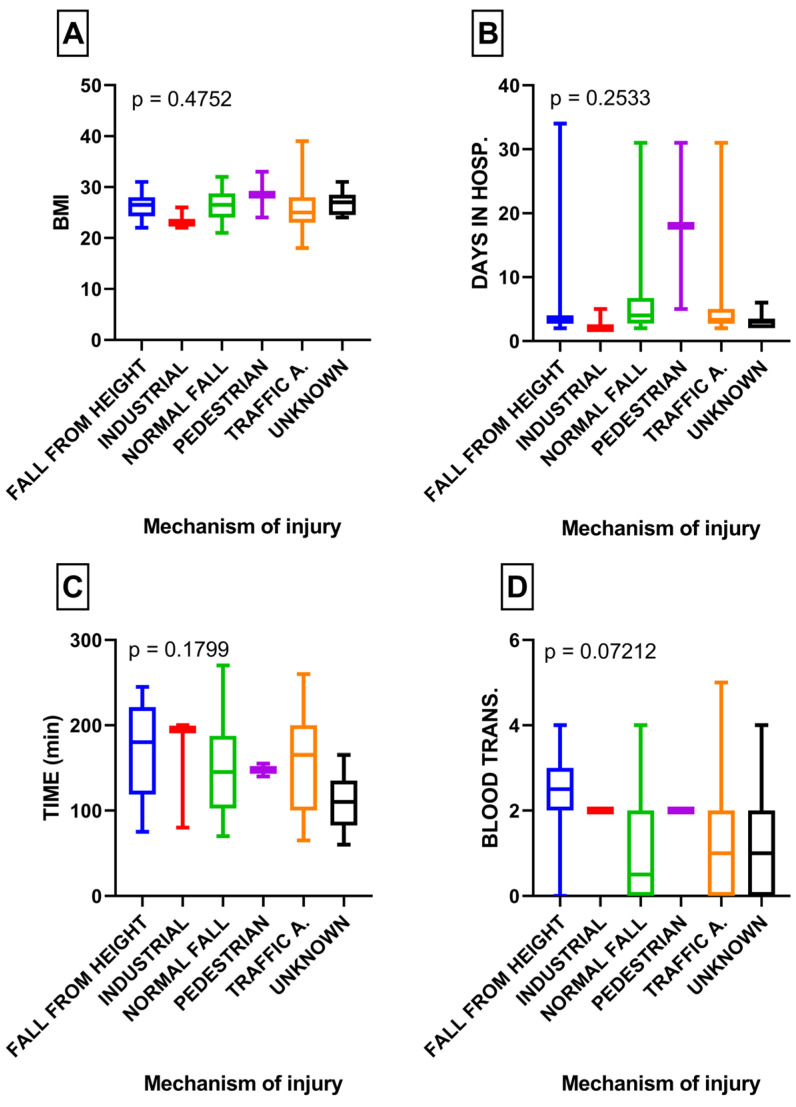
(**A**) Comparison of BMI in subgroups with various mechanisms of injury; (**B**) comparison of length of hospital stay in subgroups with various mechanisms of injury; (**C**) comparison of surgery duration in subgroups with various mechanisms of injury; (**D**) comparison of blood transfusion in subgroups with various mechanisms of injury.

The classification based on the mechanism of injury did not reveal any statistically significant differences when analyzing BMI (*p* = 0.47), length of hospital stay (*p* = 0.25), surgery duration (*p* = 0.17), and blood transfusion (*p* = 0.07) (Figure 2A–D).

However, the variable of blood transfusion approached significance, with falls from height showing a tendency toward increased blood transfusion requirements (*p* = 0.07) (Figure 2D).

**Figure 3:**
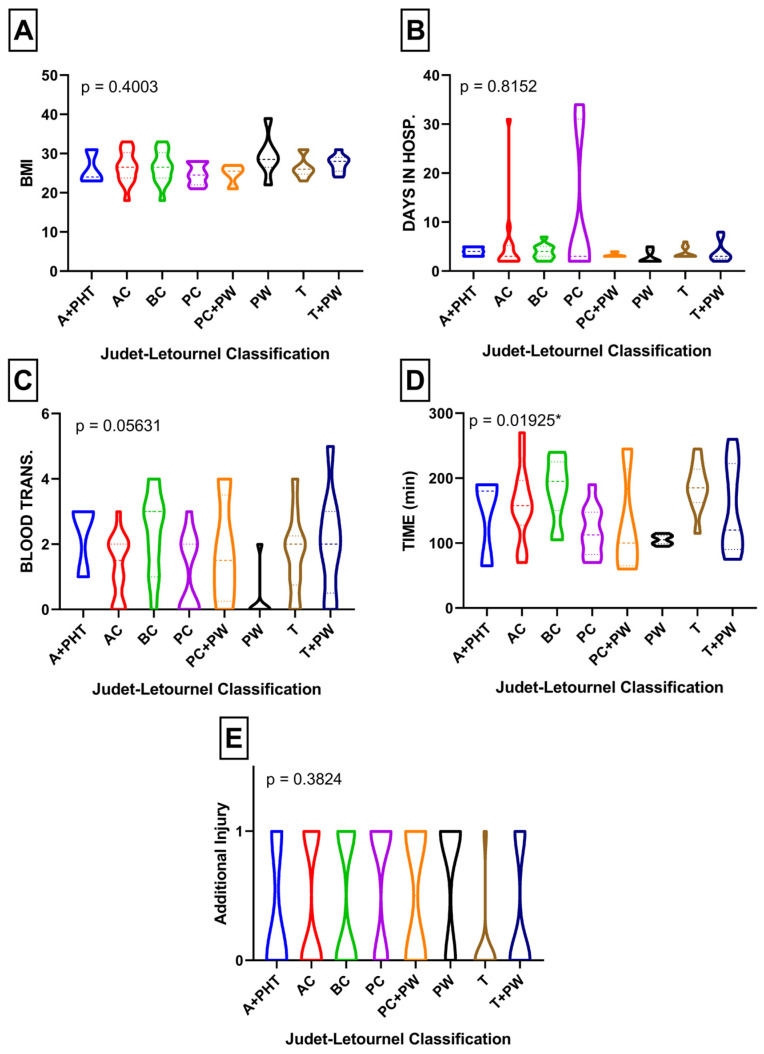
(**A**) Comparison of BMI according to Judet and Letournel subdivisions; (**B**) comparison of length of hospital stay according to Judet and Letournel subdivisions; (**C**) comparison of blood transfusion according to Judet and Letournel subdivisions; (**D**) comparison of surgery duration according to Judet and Letournel subdivisions; (**E**) comparison of additional injury in specific Judet and Letournel subdivisions. * Statistically significant result.

When analyzing the population according to the Judet and Letournel classification, no significant differences were observed in BMI (*p* = 0.40), length of hospital stay (*p* = 0.81), blood transfusion (*p* = 0.056), and concomitant injury (*p* = 0.38) within certain subgroups. However, posterior wall stabilization demonstrated the lowest rate of blood transfusion and approached statistical significance (*p* = 0.056) (Figure 3A–E). Notably, the posterior wall stabilization group exhibited the shortest surgery duration (*p* = 0.01) (Figure 3D).

**Figure 4:**
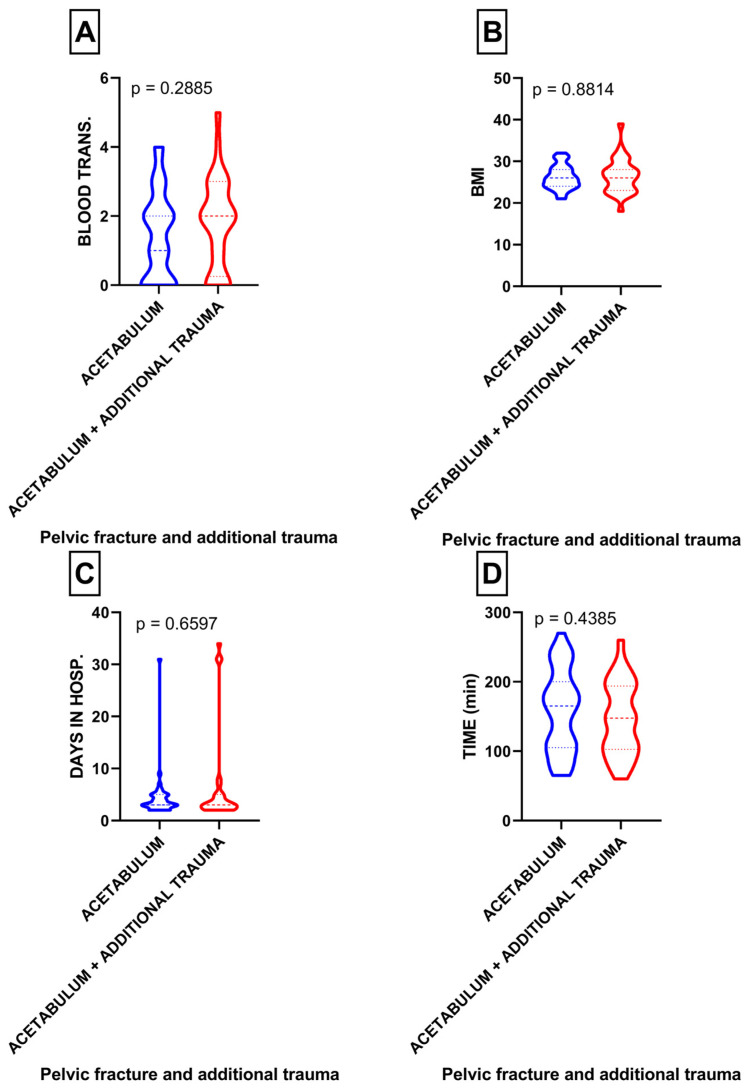
(**A**) Comparison of blood transfusion in patients with acetabulum fractures with or without concomitant trauma; (**B**) comparison of BMI in patients with acetabulum fractures with or without concomitant trauma; (**C**) comparison of length of hospital stay in patients with acetabulum fractures with or without concomitant trauma; (**D**) comparison of surgery duration in patients with acetabulum fractures with or without concomitant trauma.

Within the studied cohort, 28 patients had an additional injury. When analyzing the relationship between acetabular fractures and the additional injuries involving the spine, head, chest, abdomen, and upper/lower limbs, no significant differences were found in terms of blood transfusion (*p* = 0.28), BMI (*p* = 0.88), length of hospital stay (*p* = 0.65), and surgery duration (*p* = 0.43) (Figure 4A–D).

**Figure 5:**
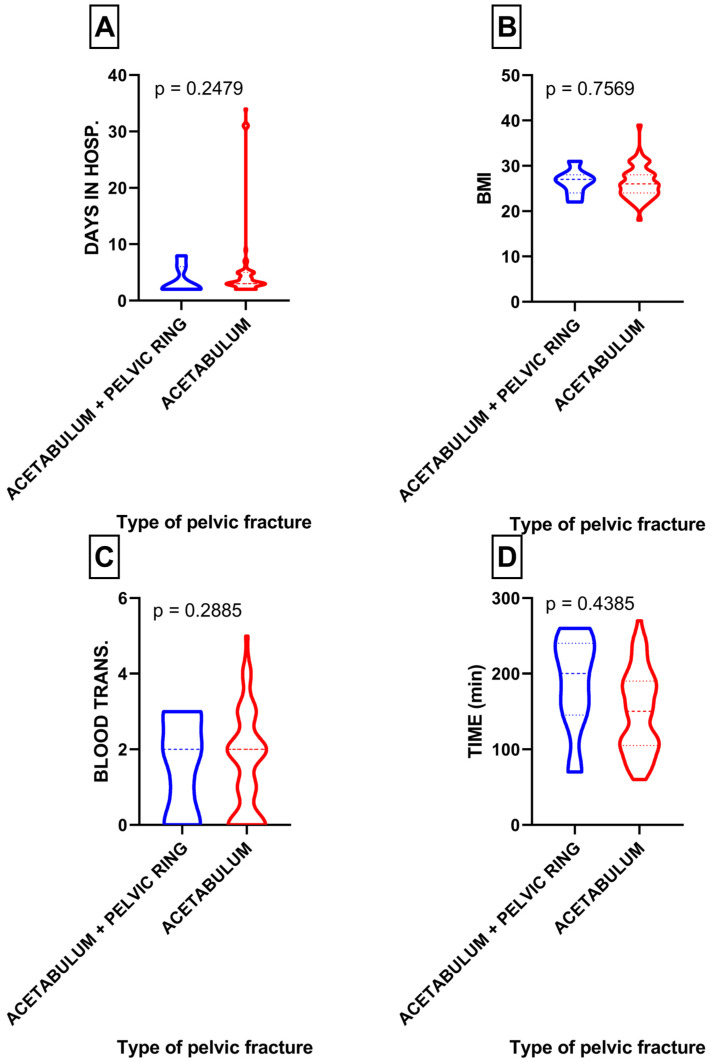
(**A**) Comparison of length of hospital stay in patients with acetabulum fractures with or without pelvic ring fracture; (**B**) comparison of BMI in patients with acetabulum fractures with or without concomitant pelvic ring fracture; (**C**) comparison of blood transfusion in patients with acetabulum fractures with or without concomitant pelvic ring fracture; (**D**) comparison of surgery duration in patients with acetabulum fractures with or without pelvic ring fracture.

Among the participants, only seven patients had an additional pelvic ring injury, specifically LC II, LC III, and APC II injuries. When comparing exclusively acetabulum fractures with acetabulum fractures accompanied by a concomitant pelvic ring injury, no statistically significant differences were observed in terms of blood transfusion (*p* = 0.28), surgery duration (*p* = 0.43), BMI (*p* = 0.75), and length of hospital stay (*p* = 0.24) between these two groups (Figure 5A–D).

**Figure 6:**
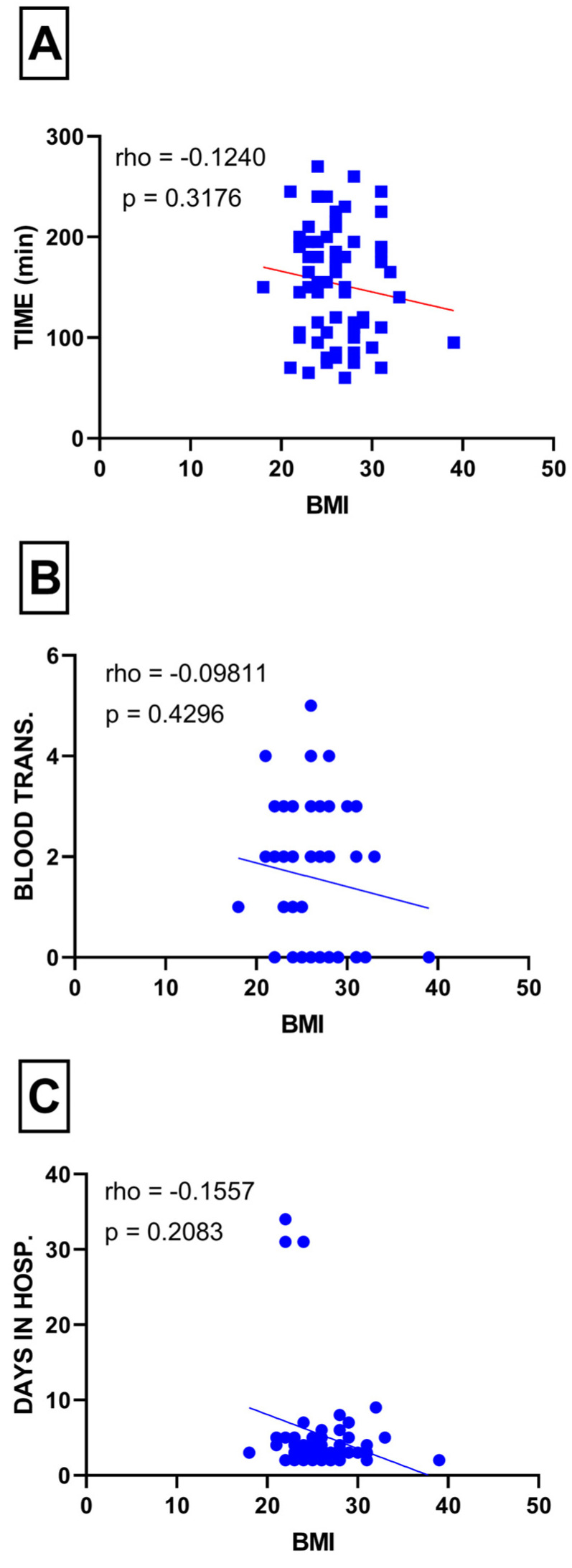
(**A**) Correlation between surgery duration and BMI; (**B**) correlation between blood transfusion and BMI; (**C**) correlation between length of hospital stay and BMI.

The Spearman rho correlation analysis between BMI and surgery duration (*p* = 0.31), blood transfusion (*p* = 0.42), and length of hospital stay (*p* = 0.20) did not reveal a statistically significant relationship (Figure 6A–C).

## 4. Discussion

The present study found no statistically significant differences among the examined groups of patients with pelvic fractures in terms of BMI, surgery duration, length of hospital stay, and blood transfusion. However, two notable findings approached statistical significance. Firstly, patients who had experienced a fall from height while sustaining a pelvic fracture required a higher number of blood transfusions (2.3 units) than those with other mechanisms of injury (Figure 2D), which was close to achieving statistical significance (*p* = 0.07). This finding is likely attributed to the high prevalence of concomitant injuries associated with falls from height, which are known to be high-energy traumas [18].

Secondly, patients undergoing posterior wall stabilization required a significantly lower number of blood transfusions than those with other specific pelvic injuries (0.33 units per patient), approaching statistical significance (*p* = 0.056). This result is consistent with the findings of Magnussen et al., who demonstrated that patients with posterior wall fractures required fewer blood transfusions than patients with other types of pelvic fractures classified according to Judet and Letournel [19]. This finding may be linked to the fact that patients in the posterior wall fracture group had the shortest mean surgery duration of 105 min among the studied population (Table 2). Additionally, the higher BMI observed in patients with posterior wall fractures could also be a contributing factor. Frisch et al. demonstrated that a higher BMI significantly reduces the likelihood of perioperative blood transfusion in total hip arthroplasty and total knee arthroplasty [20]. There are several current publications concerning BMI, blood loss, and perioperative transfusions in orthopedic surgeries, and these studies advocate for lower transfusion rates in obese and overweight patients [21,22,23,24]. Thus, our results indicating lower blood transfusion requirements in the group with the highest BMI align with these previous findings.

Furthermore, an interesting correlation was found between BMI and fracture type in the Judet and Letournel classification, similar to the findings of Waseem et al. They also reported that patients with posterior wall fractures had the highest BMI [25]. In our study, patients with posterior wall fractures had a mean BMI of 29.5, further supporting this association (Table 1).

It is not surprising that patients with both column fractures required the highest number of blood transfusions and had longer mean surgery durations (Table 1). Magnussen et al. also reported that patients with both column fractures required the highest amount of blood transfusions in their study population [19], which aligns with our findings.

In all patient groups, except for those with industrial injuries, the BMI of our study population was classified as “overweight.” Therefore, it is important to note that a high BMI has been associated with various complications in patients with pelvic fractures. Waseem et al. reported that obese patients with pelvic fractures were at a higher risk of almost every complication studied, including deep vein thrombosis, iatrogenic nerve injuries, pneumonia, and wound infection [26]. Similarly, Morris et al. observed that obese patients treated operatively for pelvic fractures experienced more complications than non-obese patients, and even among those managed conservatively, obese patients had a higher percentage of complications [27].

Some of our findings are consistent with the study conducted by Abdelrahman et al., which involved 2112 individuals with traumatic pelvic fractures [16]. In both studies, a higher proportion of males were found to have sustained pelvic fractures compared to females, with percentages of 22% vs. 78% and 23% vs. 77%, respectively [16]. Similar results were also reported by Gosh et al. and Cuthbert et al., according to which males accounted for 75% and 72% of the studied population with pelvic fractures, respectively [10,18]. 

Furthermore, our study identified similar leading causes of pelvic fractures as those reported by Abdelrahman et al., with falls from height, traffic accidents, and pedestrian incidents being the major causes [16]. Cuthbert et al. also presented comparable results, highlighting falls from height and pedestrian incidents as the two leading causes of pelvic fractures [10]. Interestingly, Abdelrahman et al. did not include falls from standing height as a specific cause of traumatic pelvic injuries requiring surgery in their study, whereas in our study, this category ranked as the second leading cause (Table 1).

Another interesting finding that contradicts other studies is the duration of hospital stay. In our studied population, the mean hospital stay for males was 4.8 days, while for females, it was 7.3 days. In contrast, Abdelrahman et al. reported a mean duration of 15 days in their population [16]. Comparable results were obtained by Gosh et al., with a mean duration of 14.4 days, although their study included both conservatively treated pelvic fractures [18].

Regarding the frequency of specific types of fractures in the Judet and Letournel classification, our analysis revealed a different pattern from that in the current literature. Trikha et al. and Fakru et al. reported the posterior wall fracture as the most common type of acetabular fracture, whereas in our studied population, the dominant injury was anterior column fractures. This finding is particularly intriguing considering that all three studies (i.e., our study, Fakru et al.’s study, and Trikha et al.’s study) identified traffic accidents as the main cause of acetabular fractures [28,29]. Providing a satisfactory explanation for these discrepancies seems challenging at this point. 

On the other hand, Vipulendran et al. found that the leading types of acetabular fractures were anterior column, anterior column + transverse, and both column fractures [30]. This finding is somewhat consistent with our study, where both column and anterior column fractures were the two leading types. Additionally, posterior column fracture was the least common type in Vipulendran et al.’s study, which correlates with our results, where it was identified as the second-to-last type [30]. However, in their studied population, falls from standing height were identified as the leading cause of pelvic injury (50%), in contrast to the studies by Trikha et al. and Fakhru et al., where traffic accidents were the main causes (77.4% and 85.3%, respectively, compared to 30% in our study). These substantial differences in fracture types among the studied populations may indicate potential problems with the accurate identification of fracture types. Further studies are needed to investigate whether this discrepancy is a significant concern.

Nevertheless, it is important to acknowledge the limitations of this study. One limitation is the restricted sample size, primarily consisting of the local population. As a retrospective study, there may be biases that could influence our results. Additionally, all data were collected by a single operative team at a single trauma center, and all procedures were performed by a surgical team consisting of two operators who regularly conducted pelvic surgeries interchangeably. Since only two operators were performing acetabular fixations in our studied population, the operators’ bias needs to be strongly addressed. The lack of a larger number of operators may have limited the breadth of our data to some extent. 

Acetabular surgeries still remain one of the most complex procedures in the orthopedic field. Therefore, only a few surgeons decide to be trained in this area. The reason why our trauma center has been performing such a substantial number of pelvic fixation surgeries is that we agree to admit and treat patients from other major trauma centers. Therefore, a potential solution to most of the limitations of our study could be to train a greater number of orthopedic surgeons in pelvic surgeries. 

Undoubtedly, further studies, particularly multicenter prospective studies, are necessary to improve our understanding and management of factors that influence appropriate treatment across a diverse population. Therefore, as a final remark, we emphasize the importance of a comprehensive evaluation of patients with pelvic fractures to achieve optimal outcomes.

## 5. Conclusions

This study provides valuable insights into pelvic fractures and their association with the investigated factors. The findings indicate that factors such as BMI, time of surgery, blood loss, and duration of hospital stay are completely correlated with the morphology of acetabular fractures, the presence of additional trauma, and the mechanism of injury. However, in the studied group, patients whose mechanism of trauma involved falling from height had an increased number of blood transfusions compared to other groups. Moreover, patients who had surgery due to posterior wall acetabulum fractures had decreased blood transfusions compared to other Judet and Letournel types of fractures. Additionally, they had the shortest duration of surgery. 

## Figures and Tables

**Table 1:** Characteristics of pelvic fractures classified according to the Judet and Letournel systems, including BMI, length of hospital stay, blood transfusion, and surgery duration.

	A + T	AC	BC	PC	PC + W	PW	T	T+P	*p*-Value
Mean BMI (kg/cm^2^)	26.0	26.5	26.5	24.8	24.8	29.5	26.4	27.4	*p* = 0.4003
Mean days in hospital (days)	4.0	5.6	3.9	11.6	3.3	2.8	3.7	3.9	*p* = 0.8152
Mean blood transfusion (units)	2.3	1.3	2.4	1.1	1.7	0.3	1.7	2.0	***p* = 0.0563**
Mean time of surgery (minutes)	145.0	159.6	183.6	119.5	126.3	105.0	186.5	152.8	***p* = 0.0193**

**Table 2:** Categorization of pelvic fractures classified according to the mechanism of injury with BMI, length of hospital stay, blood transfusion, and surgery duration.

	Fall from Height	Industrial	Fall from Standing Height	Pedestrian	Traffic Accident	Unknown	*p*-Value
Mean BMI (kg/cm^2^)	26.6	23.6	26.6	28.5	25.8	26.7	*p* = 0.4752
Mean days in hospital (days)	5.2	3.0	6.9	18.0	4.7	3.1	*p* = 0.2533
Mean blood transfusion (units)	2.3	2.0	1.1	2.0	1.37	1.2	*p* = 0.0721
Mean time of surgery (minutes)	172.8	158.3	148.0	147.5	157.6	110.0	***p* = 0.1799**

## Data Availability

No new data were created or analyzed in this study. Data sharing is not applicable to this article.

## References

[1] Morgan O., Davenport D., Enright K. (2019). Pelvic injury is not just pelvic fracture. BMJ Case Rep..

[2] Dunn E.L., Berry P.H., Connally J.D. (1983). Computed Tomography of the Pelvis in Patients with Multiple Injuries. J. Trauma Acute Care Surg..

[3] Chaumoître K., Portier F., Petit P., Merrot T., Guillon P.O., Panuel M. (2000). CT imaging of pelvic injuries in polytrauma patients. J. Radiol..

[4] Davis D.D., Foris L.A., Kane S.M., Waseem M. (2023). Pelvic Fracture. StatPearls.

[5] Holstein J.H., Culemann U., Pohlemann T. (2012). What are Predictors of Mortality in Patients with Pelvic Fractures?. Clin. Orthop. Relat. Res..

[6] Kobziff L. (2006). Traumatic pelvic fractures. Orthop. Nurs..

[7] Skitch S., Engels P.T. (2018). Acute Management of the Traumatically Injured Pelvis. Emerg. Med. Clin. N. Am..

[8] Guerado E., Medina A., Mata M.I., Galvan J.M., Bertrand M.L. (2016). Protocols for massive blood transfusion: When and why, and potential complications. Eur. J. Trauma Emerg. Surg..

[9] Coppola P.T., Coppola M. (2000). Emergency department evaluation and treatment of pelvic fractures. Emerg. Med. Clin. N. Am..

[10] Cuthbert R., Walters S., Ferguson D., Karam E., Ward J., Arshad H., Culpan P., Bates P. (2022). Epidemiology of pelvic and acetabular fractures across 12-mo at a level-1 trauma centre. World J. Orthop..

[11] Rondanelli A.M., Gómez-Sierra M.A., Ossa A.A., Hernández R.D., Torres M. (2021). Damage control in orthopaedical and traumatology. Colomb. Med..

[12] Trainham L., Rizzolo D., Diwan A., Lucas T. (2015). Emergency management of high-energy pelvic trauma. JAAPA.

[13] Rommens P.M. (2018). Focus on high energy pelvic trauma. Eur. J. Trauma Emerg. Surg..

[14] Atif M., Hasan O., Baloch N., Umer M. (2020). A comprehensive basic understanding of pelvis and acetabular fractures after high-energy trauma with associated injuries: Narrative review of targeted literature. J. Pak. Med. Assoc..

[15] Karunakar M.A., Shah S.N., Jerabek S. (2005). Body mass index as a predictor of complications after operative treatment of acetabular fractures. J. Bone Jt. Surg. Am..

[16] Abdelrahman H., El-Menyar A., Keil H., Alhammoud A., Ghouri S.I., Babikir E., Asim M., Muenzberg M., Al-Thani H. (2020). Patterns, management, and outcomes of traumatic pelvic fracture: Insights from a multicenter study. J. Orthop. Surg. Res..

[17] Buller L.T., Best M.J., Quinnan S.M. (2016). A Nationwide Analysis of Pelvic Ring Fractures: Incidence and Trends in Treatment, Length of Stay, and Mortality. Geriatr. Orthop. Surg. Rehabil..

[18] Ghosh S., Aggarwal S., Kumar V., Patel S., Kumar P. (2019). Epidemiology of pelvic fractures in adults: Our experience at a tertiary hospital. Chin. J. Traumatol..

[19] Magnussen R.A., Tressler M.A., Obremskey W.T., Kregor P.J. (2007). Predicting Blood Loss in Isolated Pelvic and Acetabular High-Energy Trauma. J. Orthop. Trauma.

[20] Frisch N., Wessell N.M., Charters M., Peterson E., Cann B., Greenstein A., Silverton C.D. (2016). Effect of Body Mass Index on Blood Transfusion in Total Hip and Knee Arthroplasty. Orthopedics.

[21] Cao G., Yang X., Yue C., Tan H., Xu H., Huang Z., Quan S., Yang M., Pei F. (2021). The effect of body mass index on blood loss and complications in simultaneous bilateral total hip arthroplasty: A multicenter retrospective study. J. Orthop. Surg..

[22] Cao G., Chen G., Yang X., Huang Q., Huang Z., Xu H., Alexander P.G., Zhou Z., Pei F. (2020). Obesity does not increase blood loss or incidence of immediate postoperative complications during simultaneous total knee arthroplasty: A multicenter study. Knee.

[23] Aggarwal V.A., Sambandam S., Wukich D. (2022). The Impact of Obesity on Total Hip Arthroplasty Outcomes: A Retrospective Matched Cohort Study. Cureus.

[24] Aggarwal V.A., Sambandam S.N., Wukich D.K. (2022). The impact of obesity on total knee arthroplasty outcomes: A retrospective matched cohort study. J. Clin. Orthop. Trauma.

[25] Waseem S., Lenihan J., Davies B.M., Rawal J., Hull P., Carrothers A., Chou D. (2021). Low body mass index is associated with increased mortality in patients with pelvic and acetabular fractures. Injury.

[26] Sems S.A., Johnson M., Cole P.A., Byrd C.T., Templeman D.C., Minnesota Orthopaedic Trauma Group (2010). Elevated body mass index increases early complications of surgical treatment of pelvic ring injuries. J. Orthop. Trauma.

[27] Morris B.J., Richards J.E., Guillamondegui O.D., Sweeney K.R., Mir H.R., Obremskey W.T., Kregor P.J. (2015). Obesity Increases Early Complications After High-Energy Pelvic and Acetabular Fractures. Orthopedics.

[28] Fakru N., Faisham W., Hadizie D., Yahaya S. (2021). Functional Outcome of Surgical Stabilisation of Acetabular Fractures. Malays. Orthop. J..

[29] Trikha V., Ganesh V., Cabrera D., Bansal H., Mittal S., Sharma V. (2020). Epidemiological assessment of acetabular fractures in a level one trauma centre: A 7-Year observational study. J. Clin. Orthop. Trauma.

[30] Vipulendran K., Kelly J., Rickman M., Chesser T. (2021). Current concepts: Managing acetabular fractures in the elderly population. Eur. J. Orthop. Surg. Traumatol..

